# “Intestinal-Type” Vulvar Adenocarcinoma: A Review of the MITO Rare Tumors Group

**DOI:** 10.3390/cancers14205171

**Published:** 2022-10-21

**Authors:** Miriam Dellino, Stefania Cicogna, Francesca Falcone, Marco Mitidieri, Roberta Mazzeo, Sandro Pignata, Giorgia Mangili, Gennaro Cormio

**Affiliations:** 1 Department of Biomedical Sciences and Human Oncology, University of Bari, 70100 Bari, Italy; 2 Clinic of Obstetrics and Gynecology, “San Paolo” Hospital, 70123 Bari, Italy; 3 Department of Obstetrics and Gynaecology, Institute for Maternal and Child Health—IRCCS “Burlo Garofolo”, 34137 Trieste, Italy; 4 Department of Gynecologic Oncology, Istituto Nazionale Tumori, IRCSS, “Fondazione G. Pascale”, 80131 Naples, Italy; 5 Azienda Ospedaliero Universitaria Città della Scienza e della Salute di Torino, Ginecologia e Ostetricia SC4, 10126 Turin, Italy; 6 Department of Medicine (DAME), University of Udine, 33100 Udine, Italy; 7 Department of Urology and Gynecology, Istituto Nazionale Tumori, IRCSS, “Fondazione G. Pascale”, 80131 Naples, Italy; 8 Obstetrics and Gynecology Unit, IRCCS San Raffaele Scientific Institute, 20132 Milan, Italy; 9 Gynecologic Oncology Unit, IRCCS Istituto Tumori Giovanni Paolo II, Department of Interdisciplinary Medicine (DIM), University of Bari “Aldo Moro”, 70121 Bari, Italy

**Keywords:** adenocarcinoma, intestinal type, vulvar cancer

## Abstract

**Simple Summary:**

Intestinal-type adenocarcinoma (VAIt) is a very rare primary neoplasia of the vulva, which must be distinguished from other more usual adenocarcinomas. A real knowledge of VAIt origin, nature, and optimum management there is not yet. Therefore, we present here a systematic review that could be a useful tool for further discussion and future clinical practice guidelines.

**Abstract:**

Intestinal-type adenocarcinoma (VAIt) represents a sporadic variant of vulvar carcinoma. It appears frequently localized to epithelial glands in the vulvar region, and it probably derives from cloacal remnants persisting in the adult. We performed a systematic review of the limited cases reported in the literature, with the intent to assess the specific peculiarities of this rare neoplasia and to state consistent management recommendations. The principal histological VAIt characteristic is that it resembles mucinous colonic carcinomas. Therefore, immunohistochemical workup, with different tumor markers including CK20, CDX2, and CK7 staining, is needed. To confirm vulvar origin, a thorough diagnostic, and radiological examination is required to rule out other primary malignancies. The gold standard of treatment for VAIt is surgery, with local excision with tumor-free margins. Lymph node staging is an option advised if the tumor size is >2 cm or if lymph node metastases are suspected on imaging. On the other hand, the role of neoadjuvant therapy is still in doubt, but a good response to adjuvant chemotherapy treatments has been described in both advanced and recurrent diseases. Sometimes, VAIt behavior can be unpredictable, with relapses even after many years, so more experiences and longer follow-up periods are needed to elucidate the best therapeutic management and its long-term prognosis.

## 1. Introduction

Approximately 90% of vulvar cancers are squamous cells in origin [1], followed by melanomas and other much less frequent neoplasms such as sarcomas, basal cell carcinomas, and adenocarcinomas [2]. Primary vulvar adenocarcinoma (VA) in particular represents a rare tumor and, consequently, its clinical behavior and prognosis are largely unknown [2]. This specific type of vulvar cancer is frequently associated with Bartholin’s glands and occurs in women during the 5°–6° decade, often clinically mimicking a Bartholin duct cyst. Rarely, VA could also arise on minor vestibular glands or Skene’s glands, on endometriosis implants, on aberrant mammary tissue, or in association with Paget’s disease [2]. Specifically, adenocarcinoma of intestinal type (VAIt) represents an extremely rare subtype of primary vulvar adenocarcinoma, and its synonyms are cloacogenic adenocarcinoma or cloacogenic carcinoma, although WHO 2020 does not recommend this terminology [3]. Therefore, in the 5th edition of the WHO classification of tumors of female reproductive organs, primary villo-glandular mucinous adenocarcinoma is described as a primary vulvar adenocarcinoma exhibiting intestinal differentiation [4]. Indeed, VAIt could be characterized by polypoid macroscopic aspects and microscopically by a villo-glandular structure composed of goblet cells or Paneth cells, characterized by intracytoplasmic mucin (similar to that of colorectal adenocarcinomas) [4]. Moreover, a conclusive diagnosis of VAIt is based on the presence of “intestinal-type” immunohistochemical phenotype and it is confirmed only when other primary neoplastic locations have been definitively excluded [4]. Because of its extremely low incidence, the best management is still debated and a unanimous consensus on treatment strategies is lacking yet. Therefore, we present here a systematic review that could be a useful tool for further discussion and future clinical practice guidelines.

## 2. Methods

**Figure 1:**
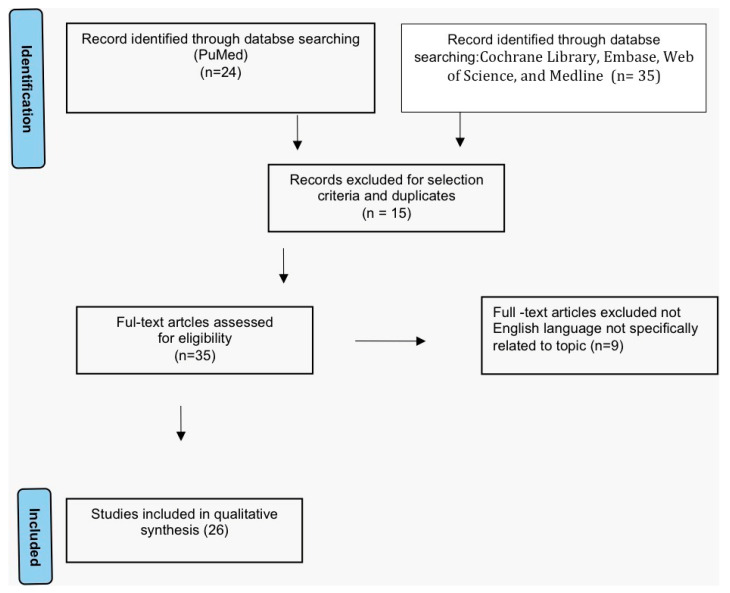
Study flow diagram: PRISMA flow diagram of identification, screening and inclusion of articles. Systematic literature reviews were selected with standard methods to be briefly presented in the article.

A systematic review of VAIt reports was performed through a literature search in the following electronic databases: PubMed, the Cochrane Library, Embase, Web of Science, and Medline databases. The articles research was performed in agreement with the Preferred Reporting Items for Systematic Reviews and Meta-Analyses (PRISMA) [5] Figure 1). The following search terms were used: “Bartholin gland”, “Adenocarcinoma” “Vulvar Cancer” and “Intestinal type”. No restrictions on the publication period were applied. Particularly, we considered articles, case series and case reports published in English. Titles and abstracts of the eligible articles were independently reviewed by two authors (M.D and S.C). Duplicates have been removed. The full texts of potentially suitable studies were independently assessed for eligibility by the two authors. Any discordance between the two sides were solved through discussion with two senior reviewers (G.C. and G.M.). Data were retrieved from articles published from 1978 (in which Tiltman and Knutzen firstly described VAIt) [6], until April 2022. Articles reporting neoplasms with mixed histology and any location in areas of the lower female genital tract other than the vulva and external genitalia (e.g., vagina) were excluded.

## 3. Results

The literature search retrieved 26 articles reporting a total of 29 cases that are listed in Table 1.

### 3.1. Epidemiology

Rare histotypes of vulvar malignancies account for less than 5% of vulvar cancer (less than 2262 estimated new cases in 2020, worldwide—GLOBOCAN 2020) [21]. Adenocarcinomas of the vulva are included in this group of neoplasms. Intestinal-type adenocarcinoma of the vulva is one of the rarest subtypes of this category, with 29 cases reported until April 2022.

### 3.2. Embryological Origin

Embryological VAIt origin still remains debated [1]. It is supposed as an embryological cloacal origin of the cervix, vagina, vulva, and perianal tissue, in which this embryonic tissue could persist [3]. In the vulva, VAIt are usually located in the vestibular portion, posterior border of introitus, and fourchette, coinciding with the area where cloacal remnants could be present [4]. Tiltman and Knutzen [6] developed the hypothesis that such misplaced vulvar remnants may undergo malignant transformation into a villoglandular adenocarcinoma of the intestinal type. These authors hypothesized that, since the lower vagina, urethra, and rectum, derived in embryologic development from the cloaca, prior to their division, a part of gastrointestinal tissue remains in the lower vagina [6]. This embryonic tissue could persist, usually in the vestibular portion corresponding to the vulvar area where cloacal residues usually remain. After that, this remnant tissue can undergo malignant transformation, analogous to primary adenocarcinoma of the colon, and subsequently become a cloacogenic adenocarcinoma [7,11]. Rodriguez et al. described this phenomenon as “Neometaplasia” that [30] defined as the existence of heterotopic differentiated tissues in tumors that are not linked embryologically to the place of origin neoplasia. Other possible theories include the presence of an ectopic intestinal epithelium or sites of intestinal metaplasia within tissues of Mullerian ductal origin [31].

### 3.3. Risk Factors and Etiopathogenesis

Some authors emphasize the role of the inflammatory process and genetic mutations in VAIt etiopathogenesis [32]. Indeed, vulvar region represents an anatomical district exposed to several environmental insults such as opportunistic and sexually transmitted infections, UV radiation and physical damage [33]. Other specific risk factors that could be connected to the development of vulvar carcinoma, comprise vulvar dystrophies, such as lichen sclerosis, smoking, and HPV infection [34], as well as in other carcinomas [19,25,35]. Various experiences concerning the relationship between HPV and vulvar lesions are reported [25,29]. On the other hand, data about the role of HPV in VAIt etiopathogenesis has not been clearly discovered yet [32]. Recently, Voltaggio et al. [29] described a series of nine HPV-related adenocarcinomas of the lower anogenital tract. Regarding VA, a single case was reported with villoglandular morphology diffuse CK20 immunoreactivity and lack of CK7 expression. Actually, in this case, the results of high-risk-HPV testing were not provided [29]. Other authors reported the association with high-risk HPV (HPV 16, detected by in situ hybridization) in one case of vestibular adenocarcinoma in situ [20,36]. However, none of the above-mentioned research reported the connection of HPV with specific vulvar cancer subtype VAIt. Only recently, Moscoso et al. [13] reported a case of VAIt, treated with wide resection, in which the presence of a low-risk HPV was detected. One of the most interesting immunohistochemical results of this case was also the evidence of p16 immuno-expression that cannot be explained with a low-risk HPV. Analogously, Houghton et al. [12], describe cases of p16 expression in cervical adenocarcinomas without the existence of oncogenic HPV. Therefore, they concluded that p16 does not represent a good marker of high-risk HPV presence in VAIt, since p16 expression can be linked to an indirect phenomenon than that due to HPV [12]. Actually, additional studies are needed to clarify the role of HPV in this kind of specific cytotype of vulvar cancer.

### 3.4. Clinical Features

Reported patient ages diagnosed with VAIt range from 31 to 92 years, with a median age of 56 years old. During vulvoscopic exams, VAIt usually appears as a unique and local exophytic lesion (rarely appears multicentric) with a median tumor diameter of 20 mm [7–60 mm] that usually arises in the vestibular portion or fourchette region [24]. Clinically, VAIt was described as an indolent mass [14], or in other cases, it was associated with pruritus, vulvar discomfort, or abnormal discharge as the most common manifestations [24]. Consequently, sometimes VAIt can mimic inflammatory processes such as recurrent Bartholin’s gland infection [37,38].

### 3.5. Pathological Examination

Most of VAIt arises from epithelium in direct continuity with the epidermis and they present usually a polypoid-like aspect and villo-glandular microscopic characters. Mucinous differentiation grade is variable and very often intestinal goblet cells or Paneth ones can be detected [24]. The mitotic rate could be differing from low to high aberrant nuclei and necrosis. Furthermore, the VAIt histology can be indistinguishable from rectum or colon similar tumors. Therefore, VAIt needs a differential diagnosis in order to be distinguished from an intestinal metastasis (which is much more frequent than a primary local lesion) [15]. Firstly, other local or systemic carcinomas should be excluded through an extensive workup, comprising systemic Positron emission tomography-computed tomography (PET-CT), colonoscopy, esophagogastroduodenoscopy (EGDS), cystoscopy, and mammography [16,38,39]. Moreover, since VAIt histologically mimics mucinous colonic carcinomas, immunohistochemical workups are needed for specific detection [37]. Unlike most Müllerian tumors of the female genital tract, VAIt often overexpresses CK20 and less frequently CK7 [15,20,22,23,29,37]. In addition, most of VAIt also show nuclear expression of the transcription factor of CDX2, which is also a specific marker of intestinal epithelium [12,15,18,20,28,29,33]. Consequently, nuclear CDX2 immunoreactivity in VAIt could represent a proof of cloacal origin. Moreover, in VAIt, the immunoreactivity for cytokeratin 7 and p16, which are characteristic for the female genital tract neoplasms was also described [8,13,22,29]. Furthermore, the literature also described in VAIt, the presence of KRAS exon 2 mutation. This genetic alteration is detected in approximately 40% of colorectal cancers and is linked with resistance to anti-epidermal growth factor receptor therapy. Due to the limited reported cases, the prognostic mean of KRAS mutations in is as unknown yet [9]. The role of lymphovascular invasion (LVSI) is not known as it has not been evaluated in most cases; in the few cases in which LVSI has been reported [7,11,13,29,37], it was present just in one patient [29] (this probably due to the fact that the carcinomatous tissue originates from embryonic residues and not from organ epithelium with a lymphatic vascularization). Only 6 out of 29 cases [6,8,10,15,38,40] presented with lymph node metastases; patients with metastatic lymph nodes had primary lesions equal to or greater than 2 cm, suggesting a prognostic role of tumor size. The staging system is the same as for other vulvar carcinomas. Specifically, the new (2021) FIGO staging for carcinoma of the vulva is applicable to VAIt [27].

### 3.6. Laboratories Analysis

To our knowledge, in the literature, there are no reported VAIt with associated increased serum tumor markers (AFP, HE4, CEA, CA125, CA15.3, and CA19.9) [10,17,26,41].

#### 3.6.1. Management

##### Neoadjuvant Chemotherapy

The use of neoadjuvant chemotherapy followed by radical surgery was recently reported as a potential option for VAIt treatment, particularly for a large tumor (5 cm) in the advanced stage (FIGO stage III) with a good response. The patient then underwent surgery with wide local excision and ipsilateral inguinal dissection (on the specimen, a residual tumor of 1.5 cm was found, with negative margins and negative lymph nodes) [22]. The rationale of neoadjuvant chemotherapy use is that it could reduce tumor volume, avoiding a demolitive surgery by limiting perioperative complications and comorbidities [22]. Specifically, the platinum-paclitaxel association is indicated in VAIt treatment, since despite significantly diverse pharmacodynamics, the combination of these drugs seems to amplify the effects of each other [42].

##### Surgery

Surgery is the cornerstone of VAIt treatment and must be individualized, based on the size of the tumor and its relationship to adjacent anatomical structures. Of the twenty-nine reported cases, only three did not undergo surgery (one for old age [10], one for advanced disease [38], and one treatment was not specified [29]). All other patients underwent surgery, ranging from local excision to radical vulvectomy with bilateral inguinal lymphadectomy. Radical vulvectomy was detected as the standard therapy until the early 2000s. In the latest years, revised surgical management has been accepted to limit surgical complications. In very small tumors (<2 cm), wide local excision appears an adequate and secure treatment [43]. In order to preserve the functionality of adjacent organs, for neoplasia that arises on the lateral or posterior of the vulva, radical local excision of the invasive lesion, with urethra and clitoris preservation, could be feasible [22,38]. Although some patients with large tumors have been recently treated with LE, short follow-ups have been reported within 12 months [7,9,13,42,43], in case of size tumors > 2 cm and according to VAIt stage, a more radical surgery could be required with a unilateral emivulvectomy with lymph node dissection or a vulvectomy with unilateral or bilateral inguinal–femoral lymphadenectomy [7,8,40,44]. In our review, lymph node staging with mono or bilateral inguinal lymphadenectomy was performed in 14 patients (less than half of the cases reported), usually in cases of large tumors or suspected enlarged lymph nodes on preoperative imaging [6,8,9,18,26,31,41]. Recently, one case of lymph node staging performed with the sentinel lymph node technique has been reported [43], presumably performed on the basis of indications for sentinel node procedure, as per the GROINSS-V study recommendations [45].

##### Adjuvant Treatment

Adjuvant chemotherapy has been proposed in some patients, especially in the case of large and/or metastatic tumors (lymph node or distant metastasis). In the literature, the use of a chemotherapy regime with mitomycin-c and 5-fluorouracil (usually indicated for colorectal primaries) was described in a case of VAIt with multiple lung metastasis, with limited response [38]. Therefore, carboplatin and taxol have been identified as the first chemotherapy choice [38]. Regarding radiotherapy, even if adenocarcinoma is not highly sensible, it is indicated particularly after surgery, in women with involved surgical margins (<5 mm) [38].

## 4. Discussion

Considering the rarity of VAIT as a neoplasm, the aim of this review is to identify its clinical and prognostic characteristics and the proposed treatments, in order to evaluate a standardized therapeutic path. VAIT affects women from the third decade onwards. The embryological origin is supposed to be linked to the persistence of residues of gastrointestinal tissue from the cloaca in the lower female genital tract. Cancerogenic risk factors are the same for other vulvar neoplasms responsible for local damage (chronic inflammation, infections, smoking); the role of HPV remains unclear. VAIt appears more frequently as an indolent mass, possibly associated with local symptoms (itching, vulvar discomfort, abnormal discharge, or simulating inflammatory processes such as recurrent bartholinitis). Histologically, they appear indistinguishable from other colorectal adenocarcinomas, with variable immunohistochemical reactivity for CDX2, CK20, and CK7. Secondaryism must be excluded, so it is mandatory to request diagnostic investigations such as PET-CT, EGDS, colonoscopy, and mammography. Data coming from reports available in the literature suggest a good response to surgical and chemotherapeutic treatments. Treatment of choice of VAIt is surgical with radical resection and tumor-free margin. Surgical procedures range from local excision to radical vulvectomy, opting for the least destructive treatment in order to reduce post-operative complications and long-term organ dysfunction [30]. Inguinal lymphadenectomy is rarely considered necessary [31]; however, lymph node staging is advised if the tumor size is >2 cm or if lymph node metastases are suspected on preoperative imaging. Adjuvant chemotherapy can be proposed in case of risk factors such as large tumors and lymph node metastases, but its effective benefit is not proven considering the limited number of cases treated. In the case of advanced VAIT, not susceptible to primary surgical treatment, neoadjuvant therapy followed by surgery could be a valuable option.

Regarding the behavior of VAIt, most reports agree that these tumors are indolent, with low growth and slight invasiveness, especially compared with other more common vulvar carcinomas such as squamous carcinoma [7,17]. Excluding one case with a short follow-up (4,5 months), the reported disease-free interval is ranging from 12 to 120 months [15]. As already mentioned, negative prognostic factors are large lesion size and lymph node involvement: tumor diameter equal to or greater than 2 cm is associated with an increased risk of lymph node metastasis, local recurrence, and a worse prognosis [6,17,18,37]; of the six cases with lymph node metastases, four died with a median PFS of 27 months [10,15,38,40]. Consequently, considering the VAIt unpredictable outcome, strict follow-up should be recommended.

## 5. Conclusions

In conclusion, VAIt is a very rare neoplasia, which must be distinguished from other more common adenocarcinomas. Real knowledge of VAIt origin, nature, and optimum management is not there yet. The meaning of HPV detection in these neoplasms is still ambiguous and more evidence is needed to explain its role in the pathogenesis of these tumors. With a diagnosis of VAIt, an intense clinical and instrumental staging is necessary to exclude a primitivity from other organs and plan a correct therapeutic management. Particularly, immunohistochemistry plays a relevant role in differential diagnosis. Treatment of VAIT is upfront surgery with local excision with curative intent lymph node staging is to be evaluated based on tumor size and suspected lymph node metastasis at preoperative imaging. Adjuvant therapy can be proposed if negative prognostic risk factors are present. In case of advanced disease, which is not suitable for primary surgery, neoadjuvant chemotherapy can be considered. Prognosis is principally associated with tumor size and lymph node status. Although VAIt generally presented an indolent progression, literature also reported several cases with a bad outcome. Therefore, pathologists and gynecologic oncologists should be engaged to standardize specific guidelines that elucidate the optimum diagnosis and treatment of this rare neoplasia. At state-of-the-art, considering the changeable VAIt behavior, personalized management and close follow-up should be provided.

## Figures and Tables

**Table 1:** Overview of cases of intestinal-type mucinous adenocarcinoma of the vulva in the literature revised until April 2022. Table Legend: MRV = modified radical vulvectomy; LNM= lymph node metastasis, LND= lymph node dissection; NED = no evidence of disease; RV = radical vulvectomy; RHV = radical hemicolectomy; WLE = wide local excision; SLN = sentinel lymph node; DOD = died of disease, CT = chemotherapy, NACT = neoadjuvant chemotherapy, RT = radiotherapy, LE = local excision, PV = partial vulvectomy, REL = relapse, NA = not available, PFS= progression-free survival.

Reference (Year)	Age (Year)	Location	Tumor Diameter (mm)	LVSI	LNM	IHC	NACT	Surgery	Adjuvant Therapy	PFS (Months)	Outcome
Tiltman et al. (1978) [5]	50	Periurethral	20	NA	Positive	NA	None	MRV + LND	None	12	NED
Kennedy et al. (1993) [7]	54	Left posterior	20	NA	Negative	CEA−, CK+	None	RV + LND	None	120	NED
	63	Posterior fourchette	15		Negative	NA		WLE		48	NED
Ghamande et al. (1995) [8]	67	Vulva	12	NA	Negative	CEA+	None	RV + LND	None	17	NED
Willen et al. (1999) [9]	57	Posterior part of vestibulum	10	NA	Negative	CEA+, CK+	None	WLE	None	26	NED
Ohno et al. (2001) [8]	92	Bartholin gland	50	NA	Positive	CEA+, CA19.9	None	NONE	RT	10	DOD
Zaidi et al. (2001) [10]	43	Posterior fourchette	50	NA	Negative	CEA+, CK+, p53+, ER−, PR−	None	MRV + LND	None	18	NED
Rodriguez et al. (2001) [11]	69	Right major labium	15	NA	Negative	CEA+, CK7+, CK20+, ER−, PR−	None	WLE	None	36	NED
Liu et al. (2003) [12]	49	Left major labium	18	absent	Negative	NA	None	WLW + LND	None	24	NED
Dube et al. (2004) [13]	58	Right major labium	15	absent	Negative	CK7+, CK20+, ER−, PR−	None	RHV + LND	None	16	NED
Dube et al. (2006) [6]	64	Hymen	7	NA	NA	CK7+, CK20-	None	WLE	None	4,5 (18 weeks)	NA
Cormio et al. (2012) [14]	59	Vestibulum	NA	NA	Positive	CK7+	None	RV + LND	None	54	DOD
	42	Vulva	10	NA	Negative	CK7+, CK20+	None	RV + LND	None	39	NED
Karkouche et al. (2012) [15]	31	Fourchette	NA	NA	NA	CK7−, CK20+	None	WLA	None	15	NED
Musella et al. (2013) [16]	57	Right major labium	50	NA	Negative	CK7-, CK20+, CEA+, CDX2+, P16+, ER-	CT	LE+ ipsilateral LND	None	17	NED
Sui et al. (2016) [17]	43	Hymen	15	NA	Negative	CK7+, p16 focal + CK20−	None	WLE	CT	24	NED
Tulek et al. (2016) [18]	62	Vulva	30	NA	Positive	CK20+, CDx2+, MUC2+, CK7+ (focal)	None	WLE	CT	36	DOD
Matsuzaki et al. (2017) [19]	68	PeriurethralVestibulum	40	NA	NA	CK7−, CK20+, CDX2+	None	WLE	None	60	NED
He et al. (2017) [20]	63	Vulva	20	NA	NA	CK20+, CDx2+, CEA+, CK7+ (focal), p16-, ER-, PR-	None	WLE	None	26	NED
Lee at al. (2017) [21]	64	Right major labium	50	absent	NA	CEA+, CK20+, CK7+, CDX 2, p53+, p16+	None	WLE	None	12	NED
Tepeoglu et al. (2018) [22]	40	Left labium minus	20	NA	Positive	CK7+ (weak), CK20+, CEA+, CDX2+	None	PV + LND	None	38	NED
Kurita et al. (2019) [23]	63	Periurethra	20		Negative	CK7−, CK20+, CDX2+	None	WLE + LND	RT	12	NED
Kaltenecker et al. (2019) [24]	53	Left major labium	60	NA	Positive	CK7−, CK20+, CEA+, p53+	None	None	CT + RT	12	DOD
Voltaggio et al. (2019) [25]	43	None	30	NA	NA	CK7, CK20	none	None	NA	NA	NA
	38	None	NA	NA	NA			None	NA	72	REL
Robinson et al. (2020) [26]	55	Vulva Bartholin gland	NA	NA	Negative	CK7+, CK20+, CDX2+, CEA+, CA19-9+.	None	WLE +SLN	NA	NA	NED
Laforga et al. (2021) [27]	45	left labium majus	30	NA	Negative	CK20+, CDX2+, CK7−, p16-	None	WLE + SLN	None	6	NED
Martín-Vallejo et al. (2021) [28]	45	Left labium majus	30	NA	Negative	CK7−, CK20+, CDX2+	None	WLE + LND	None	8	NED
Moscoso et al. (2021) [29]	66	left labium minor, vulvar fourchette	22	absent	Negative	CK7−, CK20 focal+, CDX2+, CEA+, p16+	None	WLE + LND	None	12	NED

## References

[1] Siegel R.L., Miller K.D., Fuchs H.E., Jemal A. (2022). Cancer statistics, 2022. CA Cancer J. Clin..

[2] Kurman R.J., Ellenson L.H., Ronnett B.M. (2011). Blaustein’s Pathology of the Female Genital Tract.

[3] Di Donato V., Casorelli A., Bardhi E., Vena F., Marchetti C., Muzii L., Benedetti Panici P. (2017). Bartholin gland cancer. Crit. Rev. Oncol. Hematol..

[4] Höhn A.K., Brambs C.E., Hiller G.G.R., May D., Schmoeckel E., Horn L.C. (2021). 2020 WHO Classification of Female Genital Tumors. Geburtshilfe Frauenheilkd.

[5] Page M.J., McKenzie J.E., Bossuyt P.M., Boutron I., Hoffmann T.C., Mulrow C.D., Shamseer L., Tetzlaff J.M., Akl E.A., Brennan S.E. (2021). The PRISMA 2020 statement: An updated guideline for reporting systematic reviews. Syst. Rev..

[6] Tiltman A., Knutzen V. (1978). Primary adenocarcinoma of the vulva originating in misplaced cloacal tissue. Obstet. Gynecol..

[7] Lee I.H., Kim M.K., Lee Y.K., Hong S.R., Lee K.H. (2017). Primary mucinous adenocarcinoma of the vulva, intestinal type. Obstet. Gynecol. Sci..

[8] Tepeoglu M., Uner H., Haberal A.N., Ozen O., Kuscu E. (2018). Cloacogenic Adenocarcinoma of the Vulva: A Case Report and Review of the Literature. Türk Patoloji Derg..

[9] Martin-Vallejo J., Molina-Bellido P., Laforga J.B., Clemente-Perez P.A. (2021). Intestinal-type adenocarcinoma of the Bartholin gland: A case report and literature review. Gynecol. Oncol. Rep..

[10] Ohno T., Nakano T., Abe A., Sano T., Niibe Y., Oka K. (2001). Mucinous adenocarcinoma of Bartholin gland treated with radiation therapy: A case report. Jpn J. Clin. Oncol..

[11] Dube V., Lickrish G.M., MacNeill K.N., Colgan T.J. (2006). Villoglandular adenocarcinoma in situ of intestinal type of the hymen: De novo origin from squamous mucosa?. J. Low. Genit. Tract Dis..

[12] Houghton O., Jamison J., Wilson R., Carson J., McCluggage W.G. (2010). p16 Immunoreactivity in unusual types of cervical adenocarcinoma does not reflect human papillomavirus infection. Histopathology.

[13] Moscoso O., Reques A., Saco A., Castellvi J., Gomez-Hidalgo N.R., Ramon Y.C.S., Garcia A. (2022). Vulvar Adenocarcinoma of Intestinal Type: A Case Report of an Uncommon Entity. Int. J. Gynecol. Pathol..

[14] Dube V., Veilleux C., Plante M., Tetu B. (2004). Primary villoglandular adenocarcinoma of cloacogenic origin of the vulva. Hum. Pathol..

[15] Cormio G., Carriero C., Loizzi V., Gissi F., Leone L., Putignano G., Resta L., Selvaggi L. (2012). “Intestinal-type” mucinous adenocarcinoma of the vulva: A report of two cases. Eur. J. Gynaecol. Oncol..

[16] Dellino M., Carriero C., Silvestris E., Capursi T., Paradiso A., Cormio G. (2020). Primary Vaginal Carcinoma Arising on Cystocele Mimicking Vulvar Cancer. J. Obstet. Gynaecol. Can..

[17] Willen R., Bekassy, Carlen B., Bozoky B., Cajander S. (1999). Cloacogenic adenocarcinoma of the vulva. Gynecol. Oncol..

[18] Kurita T., Matuura Y., Hisaoka M., Hachisuga T. (2019). Adenocarcinoma of intestinal type of the vulva. Int. Cancer Conf. J..

[19] Tamma R., Limongelli L., Maiorano E., Pastore D., Cascardi E., Tempesta A., Carluccio P., Mastropasqua M.G., Capodiferro S., Covelli C. (2019). Vascular density and inflammatory infiltrate in primary oral squamous cell carcinoma and after allogeneic hematopoietic stem cell transplantation. Ann. Hematol..

[20] Talia K.L., Otton G., Garland S., Phillips S., Scurry J. (2017). Human Papillomavirus-Associated Adenocarcinoma In Situ of the Vestibule. J. Low Genit. Tract Dis..

[21] Sung H., Ferlay J., Siegel R.L., Laversanne M., Soerjomataram I., Jemal A., Bray F. (2021). Global cancer statistics 2020: GLOBOCAN estimates of incidence and mortality worldwide for 36 cancers in 185 countries. CA A Cancer J. Clin..

[22] Musella A., Marchetti C., Salerno L., Vertechy L., Iadarola R., Pecorella I., Panici P.B. (2013). An unexpected complete remission of advanced intestinal-type vulvar adenocarcinoma after neoadjuvant chemotherapy: A case report and a literature review. Case Rep. Obstet. Gynecol..

[23] Karkouche R., Ansart F., Terris B., Lavenu M.C., Plantier F. (2012). Multiple tubulovillous adenomas of the vulva. Am. J. Dermatopathol..

[24] He S.R., Deng W.H., Yang L., Yang K., Cui D., Liu D.G. (2017). Cloacogenic adenocarcinoma of the vulva: One new case and literature review. Eur. J. Gynaecol. Oncol..

[25] Cascardi E., Cazzato G., Daniele A., Silvestris E., Cormio G., Di Vagno G., Malvasi A., Loizzi V., Scacco S., Pinto V. (2022). Association between Cervical Microbiota and HPV: Could This Be the Key to Complete Cervical Cancer Eradication?. Biology.

[26] Ghamande S.A., Kasznica J., Griffiths C.T., Finkler N.J., Hamid A.M. (1995). Mucinous adenocarcinomas of the vulva. Gynecol. Oncol..

[27] Olawaiye A.B., Cuello M.A., Rogers L.J. (2021). Cancer of the vulva: 2021 update. Int. J. Gynecol. Obstet..

[28] Verginelli F., Pisacane A., Gambardella G., D’Ambrosio A., Candiello E., Ferrio M., Panero M., Casorzo L., Benvenuti S., Cascardi E. (2021). Cancer of unknown primary stem-like cells model multi-organ metastasis and unveil liability to MEK inhibition. Nat. Commun..

[29] Voltaggio L., McCluggage W.G., Iding J.S., Martin B., Longacre T.A., Ronnett B.M. (2020). A novel group of HPV-related adenocarcinomas of the lower anogenital tract (vagina, vulva, and anorectum) in women and men resembling HPV-related endocervical adenocarcinomas. Mod. Pathol..

[30] Rodriguez A., Isaac M.A., Hidalgo E., Marquez B., Nogales F.F. (2001). Villoglandular adenocarcinoma of the vulva. Gynecol. Oncol..

[31] Kennedy J.C., Majmudar B. (1993). Primary adenocarcinoma of the vulva, possibly cloacogenic. A report of two cases. J. Reprod. Med..

[32] Judson P.L., Habermann E.B., Baxter N.N., Durham S.B., Virnig B.A. (2006). Trends in the incidence of invasive and in situ vulvar carcinoma. Obstet. Gynaecol..

[33] Xiao X., Meng Y.B., Bai P., Zou J., Zhang Y., Nguyen T.M.B., Xiao J.G., Gao X.M., Wen B.F. (2017). Vulvar Cancer in China: Epidemiological Features and Risk Analysis. J. Cancer.

[34] Dellino M., Cascardi E., Tomasone V., Zaccaro R., Maggipinto K., Giacomino M.E., De Nicolo M., De Summa S., Cazzato G., Scacco S. (2022). Communications Is Time for Care: An Italian Monocentric Survey on Human Papillomavirus (HPV) Risk Information as Part of Cervical Cancer Screening. J. Pers. Med..

[35] Limongelli L., Cascardi E., Capodiferro S., Favia G., Corsalini M., Tempesta A., Maiorano E. (2020). Multifocal Amelanotic Melanoma of the Hard Palate: A Challenging Case. Diagn..

[36] Matsuzaki A., Saio M., Kosuge N., Aoyama H., Tamaki T., Matsumoto H., Yoshimi N. (2017). Primary Villoglandular Mucinous Adenocarcinoma of the Vulva. Case Rep. Pathol..

[37] Liu S.-H., Ho C.-M., Huang S.-H., Shih B.-Y., Lee F.-K. (2003). Cloacogenic adenocarcinoma of the vulva presenting as recurrent Bartholin’s gland infection. J. Formos. Med. Assoc..

[38] Kaltenecker B., Manos R., McCall M., Sparzak P. (2019). Intestinal-type adenocarcinoma of the vulva: A case study. Gynecol. Oncol. Rep..

[39] Dellino M., Gargano G., Tinelli R., Carriero C., Minoia C., Tetania S., Silvestris E., Loizzi V., Paradiso A., Casamassima P. (2021). A strengthening the reporting of observational studies in epidemiology (STROBE): Are HE4 and CA 125 suitable to detect a Paget disease of the vulva?. Medicine (Baltim.).

[40] Tulek F., Kahraman A., Taskin S., Yuksel S., Sertcelik A., Ortac F. (2016). Primary mucinous carcinoma of the vulva with signet ring cells deriving from the cloaca. Eur. J. Gynaecol. Oncol..

[41] Zaidi S.N., Conner M.G. (2001). Primary vulvar adenocarcinoma of cloacogenic origin. South. Med. J..

[42] Laforga J.B., Martin J. (2021). Intestinal-type mucinous adenocarcinoma of the Bartholin gland in a perimenopausal woman. A case report and review of the literature. Rev. Española De Patol..

[43] Robinson H., Karpe M., Edidi I., Fisher A., Drew Y., Ralte A., O’Donnell R.L. (2021). Enteric Type Bartholin Gland Adenocarcinoma: An Unusual Variant of a Rare Neoplasm. Int. J. Gynecol. Pathol..

[44] Sui Y., Zou J., Batchu N., Lv S., Sun C., Du J., Wang Q., Song Q., Li Q. (2016). Primary mucinous adenocarcinoma of the vulva: A case report and review of the literature. Mol. Clin. Oncol..

[45] Van der Zee A., Oonk M.H., De Hullu J.A., Ansink A.C., Vergote I., Verheijen R.H., Maggioni A., Gaarenstroom K.N., Baldwin P.J., Van Dorst E.B. (2008). Sentinel node dissection is safe in the treatment of early-stage vulvar cancer. J. Clin. Oncol..

